# Complete chloroplast genome sequence of *Acacia crassicarpa* (Fabaceae)

**DOI:** 10.1080/23802359.2021.1944365

**Published:** 2021-07-06

**Authors:** Xinjian Yue, Yuyun Yu, Wei Gao, Shipin Chen, Zebin Weng, Gongfu Ye

**Affiliations:** a College of Forestry, Fujian Agriculture and Forestry University, Fuzhou, People's Republic of China; b Forest Inventory and Planning Institute of Fujian Province, Fuzhou, People's Republic of China; c Fujian Academy of Forestry Science, Fuzhou, People's Republic of China; d Fujian Provincial Department of Forestry, Fuzhou, People's Republic of China

**Keywords:** *Acacia crassicarpa*, chloroplast genome, phylogeny, Fabaceae

## Abstract

*Acacia crassicarpa* (Fabaceae), a nitrogen-fixing tree species, is critically important for coastal protection in southeast China. In this study, we report the complete chloroplast genome sequence of *A. crassicarpa*, with a length of 176,493 bp. It contains a pair of inverted repeats (IR 39,851 bp), a large single-copy region (LSC 91,869 bp), and a small single-copy region (SSC 4,922 bp). The complete genome comprises 138 genes, including 93 protein-coding genes, 37 tRNA, and 8 rRNA genes. Our phylogenetic analysis reveals that *A. crassicarpa* is closely related to *A. podalyriifolia* and *A. dealbata*.

*Acacia crassicarpa* A.Cunn. ex Benth. 1842 (Fabaceae), a nitrogen-fixing tree species, is native to Australia, Papua New Guinea and Indonesia (Moran et al. 1989; Sulistyono et al. 2020). *Acacia crassicarpa* has been a critically important tree species for coastal protection forest in southeast China since the 1980s (Lin 2019). The wood of *A. crassicarpa* has many commercial uses, including the production of fiber, pulp, construction, shipbuilding, among others (Ling 1996). Few studies have focused on the genome of *A. crassicarpa* with only its microRNA identified to date (Liu 2015). However, comprehensive understanding of the chloroplast genome of *A. crassicarpa* is still lacking. Research into the chloroplast genome can not only improve its function, but also enhance our understanding of its biology and biodiversity. Here, we report the complete chloroplast genome sequences of *A. crassicarpa*, and reveal the phylogenetic relationships to related species in Fabaceae.

Fresh leaves of *A. crassicarpa* was collected, and dried into silica gel immediately, in Dongshan county (23°38′21.22″N, 117°24′22.17″E) in Fujian Province, China. The voucher specimen (Acr_yyy) was deposited in the Herbarium, College of Forestry, Fujian Agriculture and Forestry University (Xinjian Yue, yxinj03@126.com). Total genomic DNA was extracted from leaf tissue samples preserved in silica gel using the CTAB method (Doyle 1987). The obtained DNA was fragmented to construct a paired-end library with an insert-size of 500 bp and the genome sequencing were performed using Illumina Hiseq Xten platform, with approximately 10 GB of data generated. Illumina data were filtered by SOAPnuke (parameter: -n 0.01 -l 20 -q 0.3 -A0.25 –cutAdaptor -Q 2 -G –polyX50 –minLen 150). The chloroplast genome of *A. crassicarpa* was then assembled using the GetOrganelle pipe-line (https://github.com/Kinggerm/GetOrganelle) by recruiting plastid-like reads. Final reads were viewed and edited by Bandage (Wick et al., 2015). The assembled chloroplast genome annotation was based on the comparison with *A. dealbata* by Geneious v.11.1.5 (Kearse et al. 2012). The annotation results were drawn with the online tool OGDRAW (http://ogdraw.mpimp-golm.mpg.de/) (Marc et al. 2013).

The complete chloroplast genome sequence of *A. crassicarpa* (GeneBank accession: MW649002) was 176,493 bp in length. It contained a large single-copy (LSC) region of 91,869 bp, a small single-copy (SSC) region of 4,922 bp, and a pair of inverted repeats (IR) regions of 39,851 bp. The complete chloroplast genome was comprised of 138 genes, with 93 protein-coding genes, 37 tRNA genes, and 8 rRNA genes. The GC contents of the LSC, SSC, and IR regions individually, and of the complete genome as a whole, are 33.1%, 29.4%, 38.3%, and 35.3%, respectively.

To investigate the phylogenetic history of *A. crassicarpa*, 23 complete chloroplast genomes of Fabaceae and Magnoliaceae species were downloaded from NCBI and were aligned with MAFFT(Katoh and Standley 2013). A maximum-likelihood (ML) tree was constructed based on the 23 complete chloroplast genome sequences using the CIPRES Science Gateway web server (RAxML-HPC2 on XSEDE 8.2.12) (Miller et al. 2010) with 1000 bootstrap replicates. The phylogenetic analysis revealed that *A. crassicarpa* is closely related to *A. podalyriifolia* and *A. dealbata* (Figure 1).

**Figure 1.**
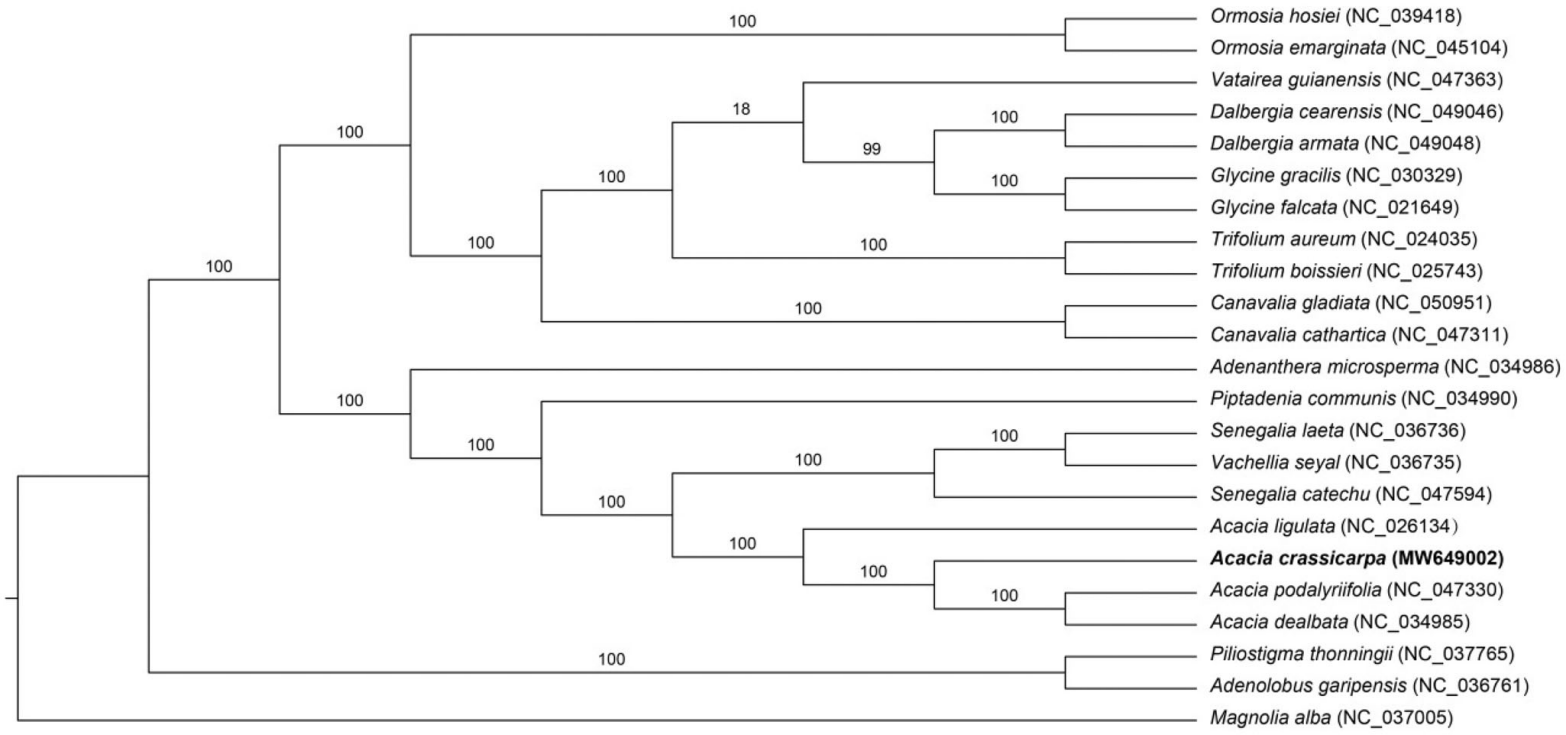
Maximum likelihood phylogenetic tree based on the complete chloroplast genome sequence of *Acacia crassicarpa* and other related species, with *Magnolia alba* representing the outgroup.

## Data Availability

The genome sequence data that support the findings of this study are openly available in GenBank of NCBI at [https://www.ncbi.nlm.nih.gov] under the accession no.MW649002. The associated BioProject, SRA, and Bio-Sample numbers are PRJNA698470, SRX10001513, and SAMN17720400 respectively.
